# Changes of GH-IGFs and its relationship with growth retardation in children with bronchial asthma

**DOI:** 10.1016/j.clinsp.2024.100385

**Published:** 2024-05-15

**Authors:** Li Li, Lihua Qiu, Junchao Xia, Yichun Xiao, Li Zhao, Haiyan Wang

**Affiliations:** a Department of Pediatrics, Ganzhou People's Hospital, Ganzhou City, Jiangxi Province, China; b Department of Pediatrics, Huaian Hospital of Huaian City, Huaian City, Jiangsu Province, China; c Department of Pediatrics, Hebei Provincial Hospital of Traditional Chinese Medicine, Shijiazhuang City, Hebei Province, China

**Keywords:** Children Bronchial Asthma, Growth Hormone, Insulin-like Growth Factor Ⅰ, Insulin-like Growth Factor Binding Protein 3, Growth Retardation

## Abstract

**Objective:**

To explore the relationship between Growth Hormone Insulin-like Growth Factors (GH-IGFs) and growth retardation in children with bronchial asthma.

**Methods:**

112 children with bronchial asthma and 50 healthy children were studied. Serum GH, IGF-1, and Insulin-like Growth Factor Binding Protein 3 (IGFBP3) were assessed by ELISA. GH-IGFs-related parameters were compared, and the correlation between the parameters and bronchial asthma severity was analyzed. The bronchial asthma group was divided into the growth retardation group and non-growth retardation group to analyze the diagnostic value of GH-IGFs in growth retardation and the relationship between GH-IGFs and growth retardation.

**Results:**

GH, IGF-1, and IGFBP3 in the bronchial asthma group were lower. GH, IGF-1, and IGFBP3 levels were decreased with the severity of bronchial asthma. GH, IGF-1, and IGFBP3 in the growth retardation group were lower than those in the non-growth retardation group. The AUC of GH-IGFs combined detection was higher than that of GH and IGFBP3 alone detection. GH < 9.27 μg/L and IGF-1 < 179.53 mmoL/L were risk factors for growth retardation in patients with bronchial asthma.

**Conclusion:**

GH-IGFs-related parameters have diagnostic value for growth retardation in children, and decreased levels of GH and IGF-1 are risk factors for growth retardation in children.

## Highlights

• GH-IGFs-related parameters.
• GH-IGFs-related parameters in patients with different severity of bronchial asthma.
• Correlation analysis of GH-IGFs-related parameters and severity of bronchial asthma.
• GH-IGFs-related parameters in patients with or without growth retardation.
• Diagnostic value of GH-IGFs-related indicators in growth retardation.
• Logistic regression analysis of GH-IGFs-related indicators and growth retardation.

## Introduction

Pediatric bronchial asthma is a heterogeneous disease characterized by chronic airway inflammation. At the onset of the disease, sporadic or diffuse wheezing rale in the expiratory phase can be heard in both lungs, and patients are often accompanied by variable expiratory airflow limitation, recurrent wheezing, shortness of breath, chest tightness, or cough. Bronchial asthma is a chronic inflammation involving a variety of inflammatory cells, which can cause airway damage, and then lead to airway smooth muscle thickening.^1^ Insulin-like Growth Factor-1 (IGF-1) is involved in the repair process of airway epithelium and alveolar injury.^2^ It has been reported in the past that the growth of airway epithelial cells can be promoted by regulating IGF-1 concentration.^3^ IGFs are a class of insulin-like peptides that promote cell differentiation and proliferation and are the main factors in the Growth Hormone (GH) - Insulin-like Growth Factors (GH-IGFs). There is a feedback regulatory system between IGF-1 and GH. GH can promote the synthesis and release of IGF-1, while IGF-1 can increase the hypothalamic somatostatin secretion and inhibit the release of GH or directly inhibit the synthesis of GH by pituitary GH cells, thereby reducing GH levels.^4^ GH-IGFs is related to the growth of children.^5^ Bone growth and development are mainly regulated by IGF-1′s endocrine function. GH can act on target cells to directly promote cell differentiation and proliferation and stimulate the production of IGF-1 in peripheral tissues.^6^ However, there is no clear report on its relationship with the growth of children with bronchial asthma. Therefore, the purpose of this study was to explore the relationship between GH-IGFs and growth retardation in children with bronchial asthma to provide a reference for the assessment of the disease and the growth of children.

## Data and methods

### Clinical data

112 children with bronchial asthma (bronchial asthma group) and 50 healthy children (control group) from January 2021 to January 2022 were studied, showing no significant difference in clinical data between the two groups (p > 0.05, Table 1).

**Table 1:** General data between the bronchial asthma group and the control group.

**Items**	**Bronchial asthma group(n=112)**	**Control group(n=50)**	**χ2/t**	**p**
Gender				
Male	62	31	0.131	0.716
Female	50	19		
Age (years)	8.92 ± 1.17	8.76 ± 1.21	0.672	0.503
Course of disease (days)	10.52 ± 2.03	10.98 ± 2.15	1.1	0.274
Maternal height (cm)	161.29 ± 4.67	162.02 ± 4.73	0.777	0.439
Paternal height (cm)	172.85 ± 5.06	172.79 ± 5.11	0.059	0.953
Family history of asthma	13	5	0.09	0.764

### Inclusion criteria

① Meeting the diagnostic criteria for bronchial asthma;^7^;

② Age < 14 years old;

③ Complete clinical data.

### Exclusion criteria

① Severe cardiac, liver and renal dysfunction;

② Congenital diseases;

③ Intrauterine growth retardation;

④ Other lung diseases;

⑤ Abnormal thyroid function;

⑥ Intracranial tumor disease.

### Bronchial severity

The bronchial asthma group was divided into grades 1, 2, 3 and 4 according to the Guideline for the diagnosis and optimal management of asthma in children.

### GH-IGFs-related parameter detection

Centrifuge treatment was conducted on the KDC-1042 high-speed centrifuge (Anhui Zhongke Zhongjia Scientific Instrument Co., Ltd.) at 3000 r/min for 10-min, with the centrifuge radius of 15 cm. Serum was separated to measure GH, IGF-1, and Insulin-like Growth Factor Binding Protein-3 (IGFBP3) on a Lab systems Dragou Wellscan K-3 microplate reader using enzyme-linked immunosorbent assay.

### Diagnostic criteria for growth retardation

The physical standard of children with developmental delay is 30 % lower than that of children in the same state.^8^

### Outcome measures

1) GH-IGFs-related parameters were compared, and the correlation between each parameter and the severity of bronchial asthma was analyzed.
2) The bronchial asthma group was divided into the growth retardation group and non-growth retardation group according to the growth conditions, and the diagnostic value of GH-IGFs related indicators for growth retardation and its relationship with growth retardation were analyzed.

### Statistical analysis

Statistics were processed using SPSS22.0 software. Enumeration data (%) were compared by χ^2^ test. Measurement data ($\bar{x}$ ± s) after the normality test were compared by *t*-test or multivariate analysis of variance. Spearman test was conducted to analyze the correlation between GH-IGFs and the severity of bronchial asthma. ROC curve was plotted to analyze the diagnostic value of GH-IGFs in growth retardation. Logistic regression was applied to analyze the relationship between GH-IGFs and growth retardation; p < 0.05 meant that the difference was statistically significant.

## Results

### GH-IGFs-related parameters

**Fig. 1:**
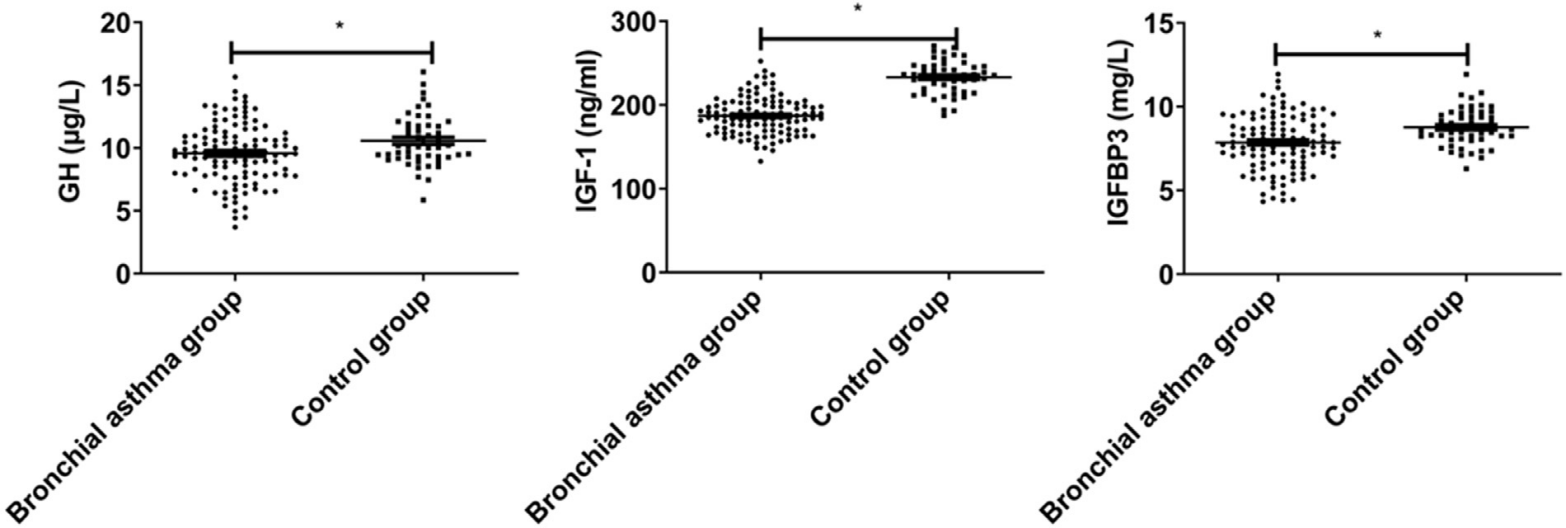
GH-IGFs-related parameters in bronchial asthma.

GH, IGF-1, and IGFBP3 levels in the bronchial asthma group were lower than those in the control group (p < 0.05, Fig. 1).

### GH-IGFs-related parameters in patients with different severity of bronchial asthma

**Fig. 2:**
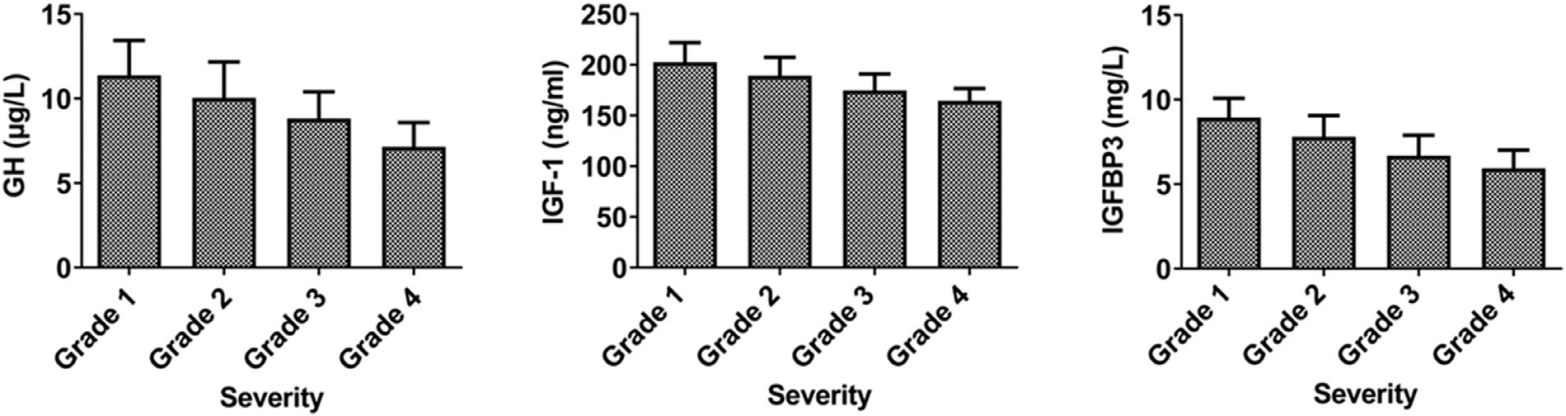
GH-IGFs-related parameters in patients with different severity of bronchial asthma.

GH, IGF-1, and IGFBP3 in patients with bronchial asthma showed a decreasing trend with the severity of the disease (p < 0.05, Fig. 2).

### Correlation analysis of GH-IGFs-related parameters and severity of bronchial asthma

**Fig. 3:**
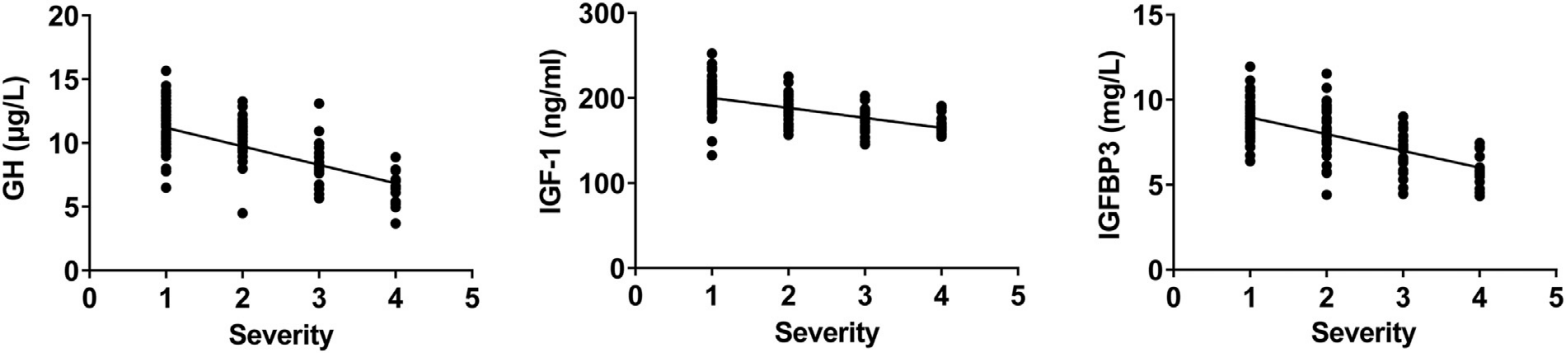
Correlation analysis between GH-IGFs-related parameters and severity of bronchial asthma.

A negative correlation was found between GH, IGF-1, and IGFBP3 levels and asthma severity (p < 0.05, Fig. 3).

### GH-IGFs-related parameters in patients with or without growth retardation

**Fig. 4:**
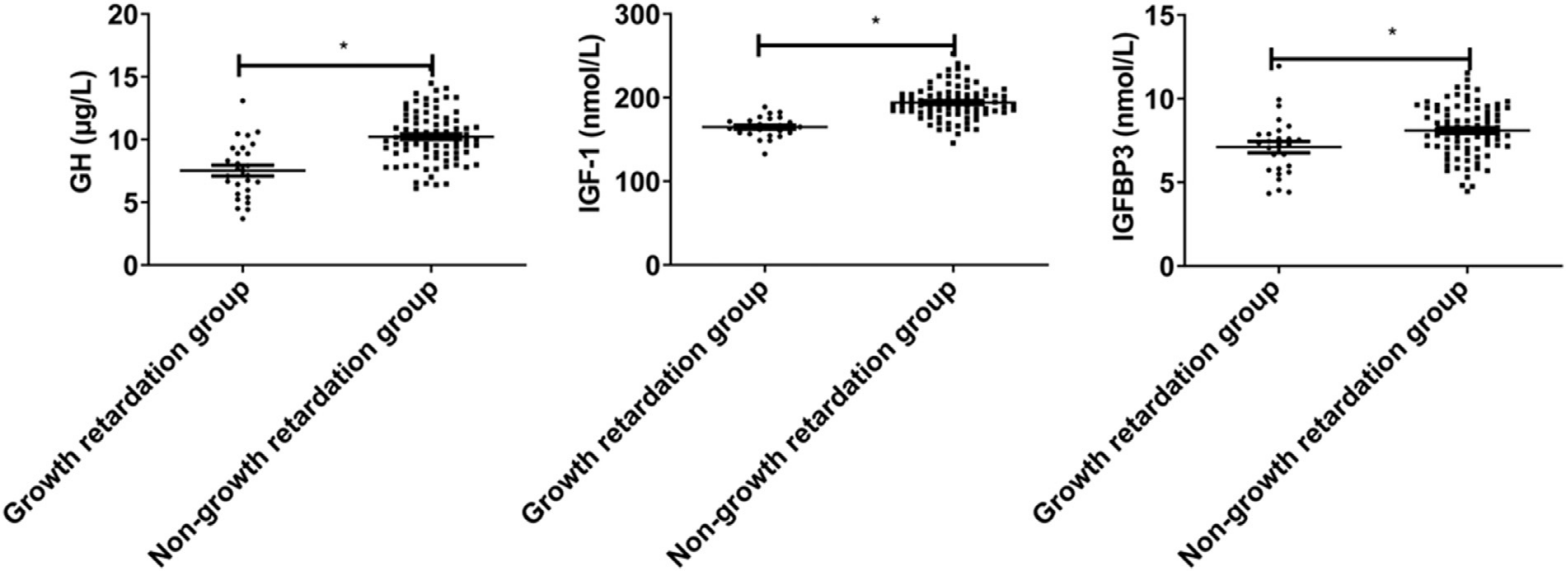
GH-IGFs-related parameters in patients with or without growth retardation.

GH, IGF-1, and IGFBP3 in the growth retardation group were lower than those in the non-growth retardation group (p < 0.05, Fig. 4).

### Diagnostic value of GH-IGFs-related indicators in growth retardation

**Fig. 5:**
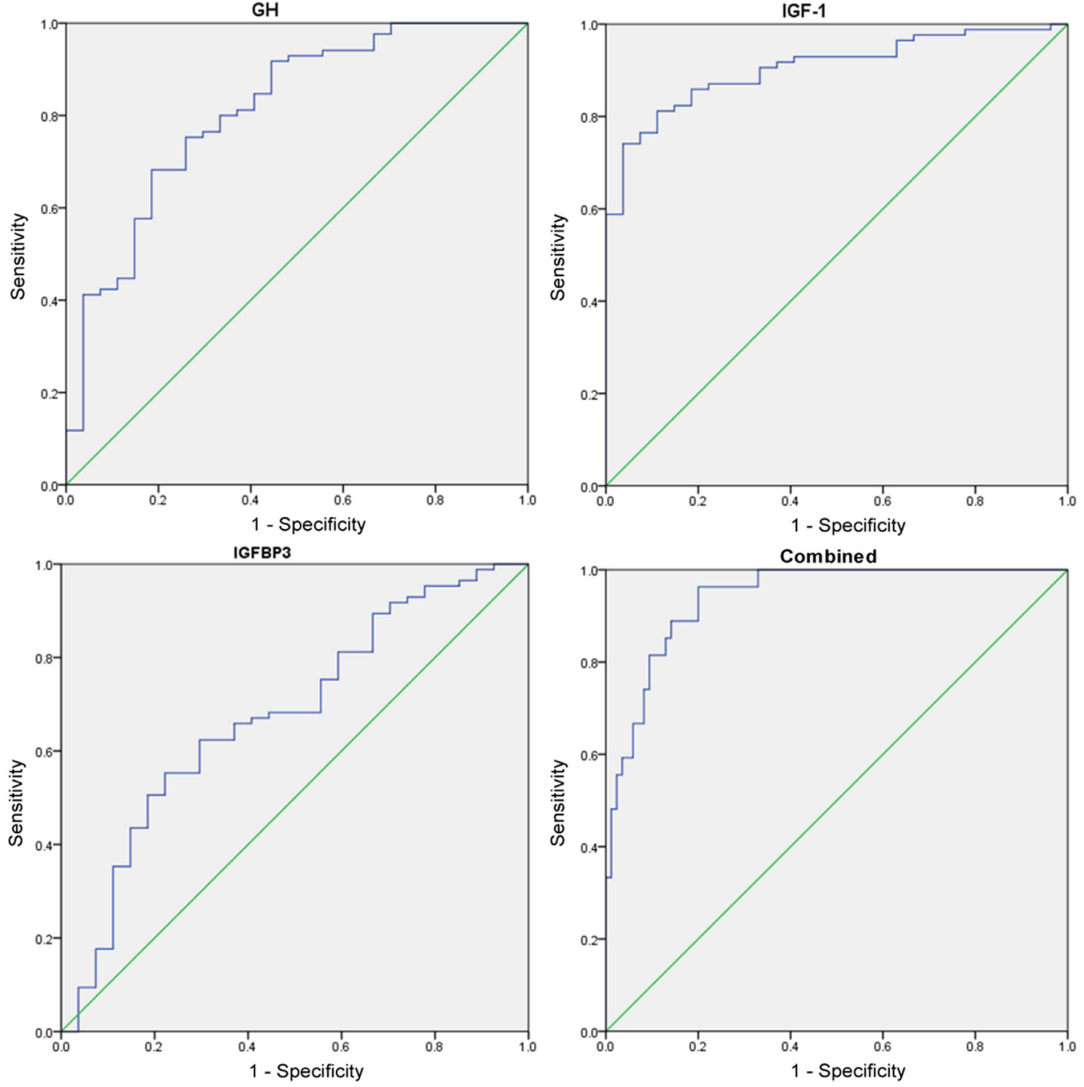
ROC curve analysis of GH-IGFs-related indicators in diagnosis of growth retardation in children with bronchial asthma. Note: Comparison between the two groups (*p < 0.05).

The AUC of the combined detection of GH-IGFs-related indicators in the diagnosis of growth retardation was greater than that of GH and IGFBP3 alone (p < 0.05, Table 2 and Fig. 5).

**Table 2:** Diagnostic value analysis of GH-IGFs system related indexes in growth retardation.

**Parameters**	**Cut-off value**	**AUC**	**SE**	**95%CI**
GH	9.27 μg/L	0.811^a^	0.049	0.715ཞ0.907
IGF-1	179.53 mmoL/L	0.905	0.028	0.850ཞ0.960
IGFBP3	8.12 mmoL/L	0.677^a^	0.06	0.559ཞ0.795
Combined		0.941	0.021	0.899∼0.982

Compared with combined.

a p < 0.05.

### Logistic regression analysis of GH-IGFs-related indicators and growth retardation

GH <9.27 μg/L and IGF-1 < 179.53 mmoL/L were risk factors for growth retardation in patients with bronchial asthma (p < 0.05, Table 3).

**Table 3:** Logistic regression analysis of GH-IGFs-related indicators and growth retardation.

**Parameters**	**β**	**SE**	**waldχ^2^**	**OR**	**95%CI**	**p**
GH	−0.614	0.197	9.714	0.541	0.368ཞ0.796	0.002
IGF-1	−0.118	0.028	17.76	0.889	0.841ཞ0.939	<0.001
IGFBP3	−0.228	0.227	1.009	0.796	0.510ཞ1.242	0.316
Constant	2.35	0.513	20.985	10.486	3.836ཞ28.659	<0.001

Assignment: GH (≥ 9.27 μg/L is 1, < 9.27 μg/L is 0); IGF-1 (≥ 179.53 mmol/L is 1, < 179.53 mmoL/L is 0); IGFBP3 (≥ 8.12 mmoL/L is 1, < 8.12 mmoL/L is 0).

## Discussion

Children's bronchial asthma is a respiratory disease that seriously affects children's physical and mental health. As this chronic inflammatory response persists, the airway is in a state of hyper-responsiveness, and the symptoms will occur repeatedly upon stimuli exposure.^9^ Inflammatory cells are involved in bronchial asthma and cause airway injury and airway smooth muscle thickening.^10^ IGF-1 is involved in the repair process of airway epithelium and alveolar injury,^11^ and regulating IGF-1 concentration can promote the growth of airway epithelial cells, suggesting that IGF-1 is involved in the process of airway epithelial cell proliferation.^12^ Blood IGF-1 can participate in the negative feedback regulation of GH by inhibiting GH expression and transcription in the pituitary gland and stimulating GH release in the hypothalamus. GH can directly act on osteoblasts to promote cell proliferation and increase type I collagen synthesis. IGF-1 can stimulate the repair of osteoblast progenitor cells, and osteoblast differentiation, and induce bone collagen synthesis. IGFBP3 is a polypeptide containing multiple amino acids, which is less responsive to GH than IGF-1. Exogenous GH can increase IGF-1 and IGFBP3 in blood, and IGF-1 infusion can decrease GH concentration, suggesting that the amount of GH in blood is closely related to that of IGF-1.^13^,^14^ In this study, GH, IGF-1, and IGFBP3 in the bronchial asthma group were lower than those in the control group, indicating the abnormal expression of GH-IGFs in children with bronchial asthma, which may be related to the involvement of IGF-1 in the repair of airway epithelium and alveolar injury. In addition, GH, IGF-1, and IGFBP3 were negatively correlated with the severity of bronchial asthma, suggesting that the severity of the disease could be assessed by detecting GH-IGFs-related parameters.

Children's is jointly regulated by the GH-IGFs. GH is secreted by adenohypophysis and regulated by growth hormone releasing hormone. Its growth-promoting effect is mainly mediated by IGF-1 and IGF-2, and IGFs secretion depends on GH. Under normal circumstances, IGF-1 level is mainly regulated by GH, and serum IGF-1 level can reflect the synthesis and release of GH. In blood circulation, IGFBP3 can combine with IGF-1 to form a complex, prolong the half-life of IGF-1 in blood vessels, and increase IGF-1 levels in blood. IGFBP3 can be degraded by IGFBP3 protease, and after degradation, the affinity between IGFBP3 and IGF-1 decreases, thereby releasing more free IGF-1 and enhancing the growth-promoting function of IGF-1.^15^,^16^

In children, GH-IGFs regulate Children's and metabolism, and the growth-promoting effect of GH is mainly mediated by IGF-1 secreted by the liver.^17^,^18^ GH binding to GHR in target cells triggers IGFs expression and secretion, wherein IGF-1 enters the tissues by binding to IGFBP, and the growth-promoting and anabolic effects of IGF-1 are triggered by IGF-I binding to IGF-IR in target organs.^19^,^20^ In this study, GH, IGF-1, and IGFBP3 in the growth retardation group were lower than those in the non-growth retardation group. The AUC of the combined detection of GH-IGFs-related indicators in the diagnosis of growth retardation was greater than that of GH and IGFBP3 alone. In addition, GH < 9.27 μg/L and IGF-1 < 179.53 mmoL/L were risk factors for growth retardation in children with bronchial asthma, indicating that decreased GH and IGF-1 could lead to growth retardation in children. The reason is that GH can stimulate peripheral tissues, especially the liver, to secrete IGF-1, which can promote organ development and activate bone anabolism. The effect of GH on growth is mediated by IGF-1, so abnormal IGF-1 can also affect Children.^21^, ^22^, ^23^

In summary, abnormal GH-IGFs-related indicators exist in children with bronchial asthma, and GH-IGFs-related indicators in children are of diagnostic value for growth and development, and decreased levels of GH and IGF-1 are risk factors for growth retardation in children.

## Availability of data and materials

The datasets used and/or analyzed during the present study are available from the corresponding author upon reasonable request.

## Ethics approval

The present study was approved by the Ethics Committee of Ganzhou People's Hospital and written informed consent was provided by all patients prior to the study start. All procedures were performed in accordance with the ethical standards of the Institutional Review Board and The Declaration of Helsinki, and its later amendments or comparable ethical standards.

## Authors’ contributions

Li Li and Lihua Qiu designed the research study. Junchao Xia and Yichun Xiao performed the research. Li Zhao and Haiyan Wang provided help and advice on the experiments. Li Li, Lihua Qiu and Haiyan Wang analyzed the data. Li Li and Lihua Qiu wrote the manuscript. Haiyan Wang reviewed and edited the manuscript. All authors contributed to editorial changes in the manuscript. All authors read and approved the final manuscript.

## Funding

Jiangxi Provincial Health and Family Planning Commission Science and Technology Program (SKJP220203187).

## Declaration of competing interest

The authors declare no conflicts of interest.
